# The Insane in Workhouse Infirmaries

**Published:** 1904-04-16

**Authors:** 


### THE INSANE IN WORKHOUSE INFIRMARIES.

While there are a great many of the insane who could be perfectly well treated in the wards of our workhouse infirmaries, always provided these wards were properly equipped and furnished, and placed under a sufficient staff of trained attendants, it is equally certain that there are numerous cases which ought not to be taken to these places at all, or, if taken there as a ready means of preventing injury to themselves or others, they should not be detained an hour longer than is necessary to permit of the requisite examination being made and certificates drawn out for removal to the county asylum. We conceive that, as a general rule, the cases suitable for workhouse treatment are those whose cure has been attempted in an asylum, but in whom chronic insanity of a harmless type has supervened. This is quite a numerous class, and Boards of Guardians would be well advised to provide suitable accommodation, as there can be no doubt that a high percentage of this class might be transferred from the asylums to the workhouse. It is far otherwise with the acute and presumably curable cases. It is the universal belief of mental specialists that the danger of chronic incurable insanity supervening on the acute stage is in direct ratio to the duration of the disease previous to the beginning of correct treatment. Every day, nay, every hour, is of importance if the reason, or sometimes the life, of the patient is to be saved.

A coroner's inquest held last week, on the body of a patient who had died in Mile End Workhouse Infirmary, has brought the question once more under public notice. In this case the patient, a man about 40 years of age, had been noticed to be strange in mind for a month, and his insanity was supposed to have been caused by loss of employment. Here was the period when asylum treatment should have been tried; but it was allowed to pass, and on March 27th he was seen dancing in front of a tram-car and dodging in and out between other vehicles. A policeman saw him and very properly took him to the workhouse infirmary; and in that institution he was kept until his death, which took place a week later. It appeared, in evidence given at the inquest, that the patient was so violent that he had to be secured to the bed by jacket-sheets and towels; and the medical officer deposed that the patient was under restraint for 140 hours. The coroner asked if that was not unusual, and the witness answered that they could do nothing with the patient. Death was due to heart failure following acute mania, and it was not thought that the restraint had accelerated death. The medical officer admitted that the accommodation provided for lunatics was not "ideal;" and when we learn that some of the attendance is left to an inmate, and that there is no day-room attached to the ward, we find no difficulty in agreeing with him.

That restraint is sometimes desirable, or even necessary for the welfare of the insane themselves is unquestionable; but it should never be employed when other and better means suffice; and above all it is not justifiable merely because it saves a certain amount of trouble to the staff of attendants, or obviates the engaging of additional paid help. The extreme rarity of its necessity may be illustrated by the fact that during the whole of the year 1902 restraint was employed once, for a few minutes only, in the Claybury Asylum, containing 2,500 patients; and it would probably have been to this East London asylum that the patient would have been removed.

The jury returned a verdict in accordance with the medical evidence; and they wished the Coroner to convey to the Guardians that in their opinion the accommodation for alleged lunatics was insufficient, and that a proper dayroom should be supplied. This rider to the verdict was not out of place, but to us the questions which need answering are, What reasons can be given for detaining an acute lunatic for a week in the infirmary, who is responsible for this detention, and are similar cases to be detained there in future? So far the evidence shows that the accommodation is not suited for the acute forms of insanity; and although we do not know of any law by which guardians can be compelled to provide the requisite accommodation, it would seem to be not more than common humanity that they should either make this provision or immediately pass acute cases on to the county asylum.

